# Supported and Non-Supported Ruthenium(II)/Phosphine/[3-(2-Aminoethyl)aminopropyl]trimethoxysilane Complexes and Their Activities in the Chemoselective Hydrogenation of *trans*-4-Phenyl-3-butene-2-al

**DOI:** 10.3390/molecules15074652

**Published:** 2010-06-30

**Authors:** Ismail Warad

**Affiliations:** Petrochemical Research Chair, Department of Chemistry, King Saud University, P. O. Box 2455, Riyadh 11451, Saudi Arabia; E-Mail: warad@ksu.edu.sa; Tel./Fax: +96-61-4675992

**Keywords:** Ru(II) complexes, hydrogenation, interphase, ether-phosphine, sol-gel, NMR

## Abstract

Syntheses of four new ruthenium(II) complexes of the [RuCl_2_(P)_2_(N)_2_] type using 2-(diphenylphosphino)ethyl methyl ether (P~O) as ether-phosphine and triphenylphosphine (PPh_3_) as monodentate phosphine ligands in the presence of [3-(2-aminoethyl)aminopropyl]trimethoxysilane as diamine co-ligand are presented for the first time. The reactions were conducted at room temperature and under an inert atmosphere. Due to the presence of the trimethoxysilane group in the backbone of complexes **1** and **2** they were subjected to an immobilization process using the sol-gel technique in the presence of tetraethoxysilane as cross-linker. The structural behavior of the phosphine ligands in the desired complexes during synthesis were monitored by ^31^P{^1^H}-NMR. Desired complexes were deduced from elemental analyses, Infrared, FAB-MS and ^1^H-, ^13^C- and ^31^P-NMR spectroscopy, xerogels **X1 ** and **X2** were subjected to solid state, ^13^C-, ^29^Si- and ^31^P-NMR spectroscopy, Infrared and EXAF. These complexes served as hydrogenation catalysts in homogenous and heterogeneous phases, and chemoselective hydrogenation of the carbonyl function group in *trans*-4-phenyl-3-butene-2-al was successfully carried out under mild basic conditions.

## 1. Introduction

Reduction of aldehydes and ketones to the corresponding alcohols is a core technology in fine chemicals synthesis, particularly for pharmaceuticals, agrochemicals, flavors and fragrances, which requires a high degree of stereochemical precision [1,2,3]. Asymmetric hydrogenations of C=C, C=O, and C=N functionalities have found important applications in organic synthesis and in the fine chemical business [3,4,5,6,7,8]. A high turnover frequency (TOF) can be obtained by designing suitable molecular catalysts and reaction conditions. Preferential reduction of a C=O function over a coexisting C=C linkage is an important and difficult task. Although there are many examples of highly efficient catalysts for olefin and ketone reduction, imine hydrogenation is still a challenge in terms of both the turnover frequency and the lifespan of the active catalyst [9,10,11,12,13,14,15,16,17,18,19,20,21,22,23,24,25]. One of the best transition-metal complexes for ketone hydrogenation that has been discovered is the chiral Ru(II)–diphosphine/1,2-diamine complex, which was developed by Noyori [3]. This system was found to be active in the chemoselective hydrogenation of carbonyl functional groups in the presence of olefins and in the reduction of imines [5,6,7,8]. The immobilization of metal complexes enables the longterm use of expensive or toxic catalysts and provides a clean and straightforward separation of the product [26,27,28,29,30,31,32,33]. 

It is interesting to investigate the chemical properties and structures of new ruthenium(II) complexes containing C=C functional groups in the backbone of the phosphine ligand and indirect to the phosphorus atom [1,1-bis(diphenylphosphinomethyl)ethane, dppme] ligands to see how these properties are related to the chemical behavior of complexes by monitoring any changes by ^31^P{^1^H}- NMR spectroscopy [21].

Phosphorus–oxygen hemilabile ligands like 2-(diphenylphosphino)ethyl methyl ether ( P~O), reacts with various metals of catalytic relevance due to their ability to act as both a chelate ligand, stabilizing the metal complex, and a monodentate ligand providing a free coordination site for an incoming substrate (through the labilization of the weakly bonded oxygen atom) [12,13,14,15,16,17,18,19].

By the introduction of T-functionalized into the diamine ligands coordinated complexes, these complexes can be easily immobilized as atypical interphase to a polysiloxane matrix by sol-gel process [9,10,17,26,27,28,29,30,31,32,33]. In such interphases the stationary phase (comprising active centers, polymer and spacer) and a mobile component (gas, liquid or dissolved reactants) penetrate each other on a molecular scale without forming a homogeneous phase [26,27,28,29,30,31,32,33]. When such interphases are provided with a swellable polymer, they may imitate homogeneous conditions as the active centers become highly mobile, simulating the properties of a solution [9,10,17,26,27,28,29,30,31,32,33]. 

Recently we have synthesized a number of ruthenium(II) complexes of the [RuCl_2_(P)_2_(N)_2_] type using both mondentate or bidentate phodsphine and amine ligands, and these complexes were tested as hydrogenation catalysts for functionalized carbonyl compounds. Our ongoing research interest is in synthesizing supported and non-supported phosphine/diamine Ru(II) complexes, then examining their activity for catalytic hydrogenation in both homogenous and heterogeneous phase [9,10,17].

In this work a set of ruthenium(II)/phosphine/diamine complexes were made available by using monodentate triphenylphosphine and monodetate/bidentate ether-phosphine ligands in the presence of the [3-(2-aminoethyl)aminopropyl]trimethoxysilane as diamine co-ligand. The presence of Si(OEt)_3 _anchoring groups in the backbone of the these complexes enabled the immobilization process through a simple sol-gel reaction using Si(OEt)_4_ as cross-linker. The desired complexes served as catalysts for selectivity hydrogenation of *trans*-4-phenyl-3-butene-2-al in both homogenous and heterogeneous phases under mild conditions.

## 2. Results and Discussion

### 2.1. Ruthenium(II) complexes **1** and **2** synthetic investigation and structural behavior

**Scheme 1:**
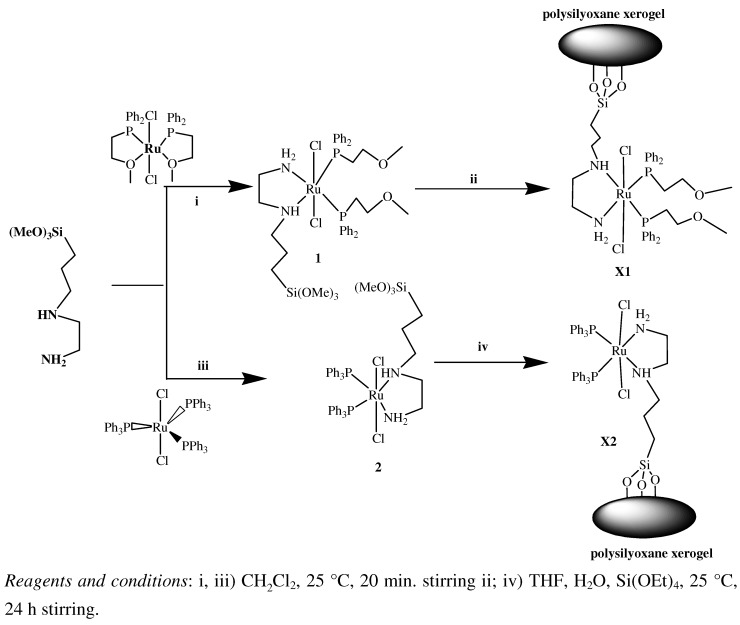
The synthetic route to prepare complexes **1-2** and xerogels **X1** and **X2**.

Treating each of Cl_2_Ru(P^⏜^O)_2_ and Cl_2_Ru(PPh_3_)_3_ individually with an equivalent amount of [3-(2-aminoethyl)aminopropyl]trimethoxysilane in dichloromethane resulted in the formation of complexes **1** and **2**, respectively, as shown in Scheme 1.

High melting yellow powders were obtained in very good yields. These complexes are soluble in chlorinated solvents such as chloroform or dichloromethane and insoluble in polar or non-polar solvents like water, methanol, diethyl ether and *n*-hexane. The structures of the desired complexes have been deduced from elemental analysis, infrared spectroscopy, FAB-mass spectrometry, ^1^H-,^ 13^C{^1^H}− and ^31^P{^1^H}−NMR spectroscopy data.

**Figure 1:**
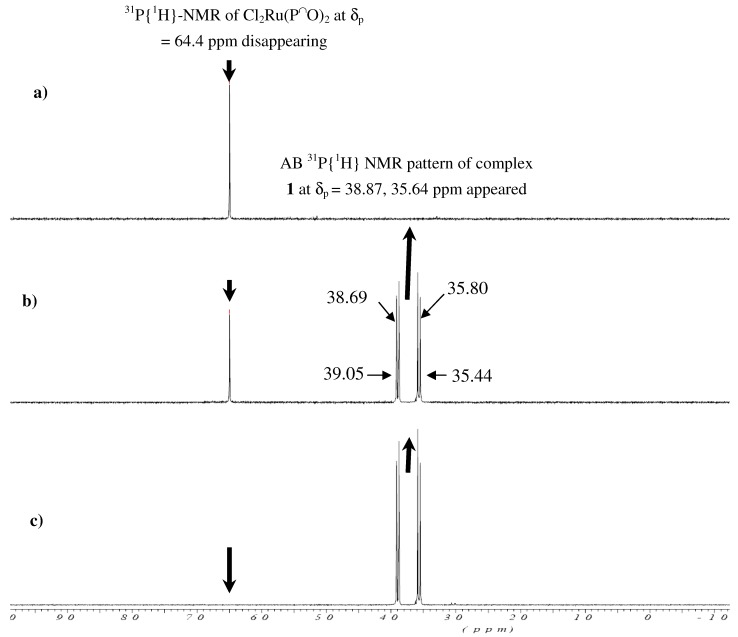
Time-dependent ^31^P{^1^H}-NMR spectroscopic of Cl_2_Ru(P^⏜^O)_2 _at δ_p _= 64.4 ppm mixed with equivalent of diamine co-ligand in CDCl_3_ in the NMR tube to produce complex **1** at δ_p _= 38.87, 35.64 ppm a) before co-ligand addition, b) the first shot ~ 0.5 min. and c) the second shot ~1 min. after the co-ligand addition.

The stepwise formation of complex **1** is monitored by ^31^P{^1^H}-NMR spectroscopy, in an NMR tube experiment, where addition of [3-(2-aminoethyl)aminopropyl]trimethoxysilane to a CDCl_3_ solution containing Cl_2_Ru(P^⏜^O)_2_ complex as starting material leads to the disappearance of the red color of the Cl_2_Ru(P^⏜^O)_2_ complex and the singlet of this complex at δ_p _= 64.40 ppm and the appearance of a new AB ^31^P{^1^H}-NMR pattern at δ_p _= 38.87, 35.64 ppm due to the formation of complex **1** with a *trans*-Cl_2_Ru(dppme)NN formula, together with the appearance of the yellow color of the latter, confirming the hemilabile cleavage of 2Ru-O to form the 2Ru-N complex **1** in a very short time and without side products, as seen in Figure 1.

The weak ruthenium-oxygen bonds in bis(chelate)ruthenium(II) complexes of the Cl_2_Ru(P^⏜^O)_2_ type are easily cleaved by an incoming molecule such as the [3-(2-aminoethyl)aminopropyl]-trimethoxysilane co-ligand. Due to the hemilabile character the oxygen donor in the ether-phosphine ligand is regarded as an intramolecular solvent impeding decomposition of the complex by protection of vacant coordination sites, which accelerates and stabilizes the synthesis of complex **1** without any side products.

### 2.2. Oxidative decomposition of complexes **1** and **2** by oxygen or H_2_O_2_

**Figure 2:**
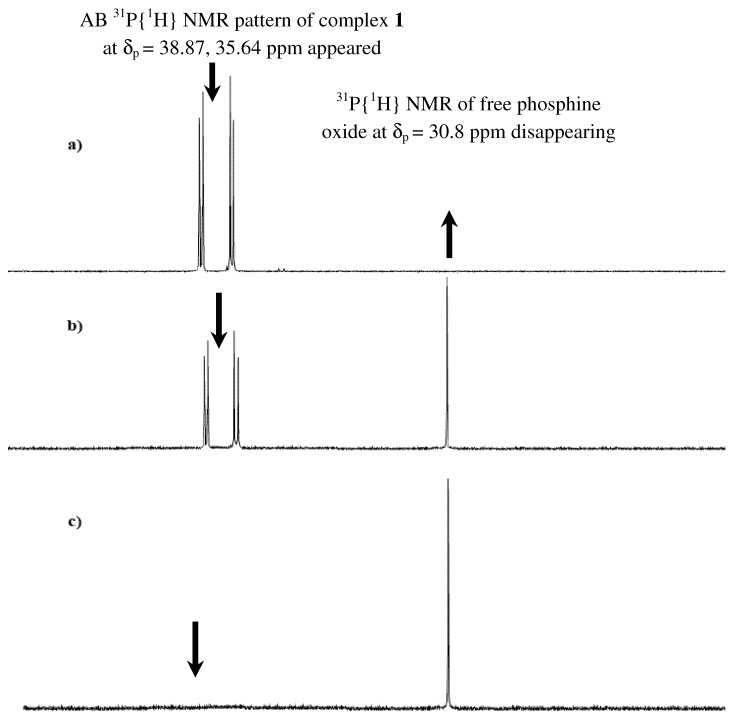
Time-dependent ^31^P{^1^H} NMR spectroscopic of complex **1** at δ_p _= 38.9, 35.6 ppm in an open atmosphere a) fresh synthesis b) after 25 min. c) after 60 min.

The desired complexes showed some sensitivity toward oxygen in solution, and the colour changed from yellow to green if oxygen allowed to enter the reaction, or if the reactions were carried out in an open atmosphere. To examine the stability of complex **1** toward oxygen, 0.10 g was dissolved in 20 mL of dichloromethane in an open atmosphere, and several samples were taken over time and subjected to ^31^P{^1^H}-NMR. The spectra showed that complex **1** (indicated by peaks at δ_p _= 38.9, 35.6 ppm) was decomposed to form phosphine oxide [Ph_2_P(=O)CH_2_CH_2_OCH_3_] with δ_p _= 30.8 ppm and other green oily ruthenium complexes in around one hour, as shown in Figure 2. These complexes are mostly free of phosphine or paramagnetic ruthenium(III) species because nothing except the phosphine oxide were detected by ^31^P{^1^H}-NMR.

Under identical conditions complex **2** displayed more stability toward oxygen compared with complex **1**, 2 hours are required to decompose the same quantity of complex **2** completely, which confirmed that phosphine complexes were more stable than the ether-phophine ones. The oxidatative decomposition processes of complexes **1** and **2** were accelerated by addition of H_2_O_2_ as another oxidizing agent. By adding very small testing drop of H_2_O_2_ to solution of the same quantity of complexes **1** or **2** the colors changed to green immediately. In general, only seconds were required to ensure the complete decomposition of the more stable phosphine complex **2** when 1:1 (mole:mole) of [H_2_O_2_:complex] were mixed. 

### 2.3. Ruthenium(II) complexes1 and2 and synthetic investigation of xerogels **X1** and **X2**

Complexes **1 ** and **2** are very important in interphase chemistry. They can be converted as primary complexes to prepare stationary phases *via* the sol gel technique in order to support complexes that transform the system from homogenous to heterogeneous phase or interphase catalysts [33,34,35]. Complexes **1** and **2** were subjected to a typical sol-gel polymerization process at room temperature in the presence of 10 equivalents of Si(OEt)_4_ as cross-linker using methanol/THF/water to prepare polysiloxane xerogels **X1** and **X2**, as shown in Scheme 1. Due to the poor solubility of the xerogels**X1 ** and **X2** they were subjected to solid state measurements like NMR, IR and EXAF. 

### 2.4. ^31^P-NMR investigation of complexes **1** and **2** and Xerogels **X1** and **X2**

**Figure 3:**
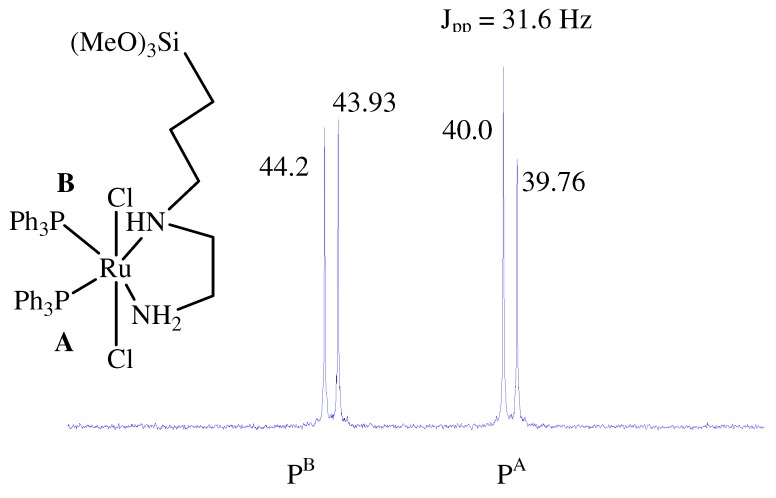
^31^P{^1^H}-NMR spectroscopic data of complex **2** at δ_p _= 39.9, 44.1 ppm and their coupling constant *J*_pp_ = 31.6 Hz.

The use of an asymmetric diamine co-ligand such as [3-(2-aminoethyl)aminopropyl]trimethoxy-silane caused the loss of the *C2 *axis, resulting in a splitting of the ^31^P{^1^H}-NMR resonances of **1**, **2**, **X1** and **X2** into AB patterns [14,27]. The phosphorous chemical shifts and the ^31^P -^ 31^P coupling constants (*J*pp = 30–36 Hz) of the desired complexes suggest that the phosphine ligand was positioned *trans *to the diamine, with *trans *dichloro atoms, to form the kinetically favored *trans*-Cl2Ru(II) isomer, as seen in Figure 3. 

### 2.5. ^1^H and ^13^C-NMR investigations

**Figure 4:**
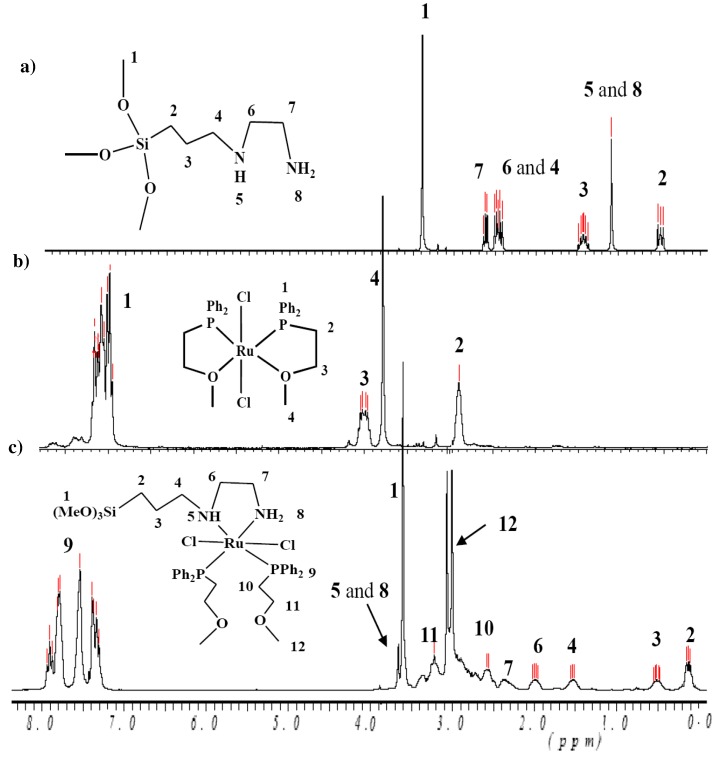
^1^H-NMR of: a) free ligand [3-(2-aminoethyl)aminopropyl]trimethoxysilane; b) complex Cl_2_Ru(P^⏜^O)_2_ starting material and c) complex **1** in CDCl_3_ at room temperature.

In the ^1^H-NMR spectra of complexes **1** and **2** characteristic sets of signals were observed, which are attributable to the phosphine as well as [3-(2-aminoethyl)aminopropyl]trimethoxysilane ligands. Their assignment was supported by a free ligand ^1^H-NMR study. The integration of the ^1^H resonances confirms that the phosphine to diamine ratios are in agreement with the compositions of the desired complexes. A comparison the ^1^H-NMR spectra of the free diamine co-ligand [3-(2-aminoethyl)amino-propyl]trimethoxysilane, individual starting material Cl_2_Ru(P^⏜^O)_2_ complex and complex **1** (after mixing Cl_2_Ru(P^⏜^O)_2_ with [3-(2-aminoethyl)aminopropyl]trimethoxysilane) is shown in Figure 4.

**Figure 5:**
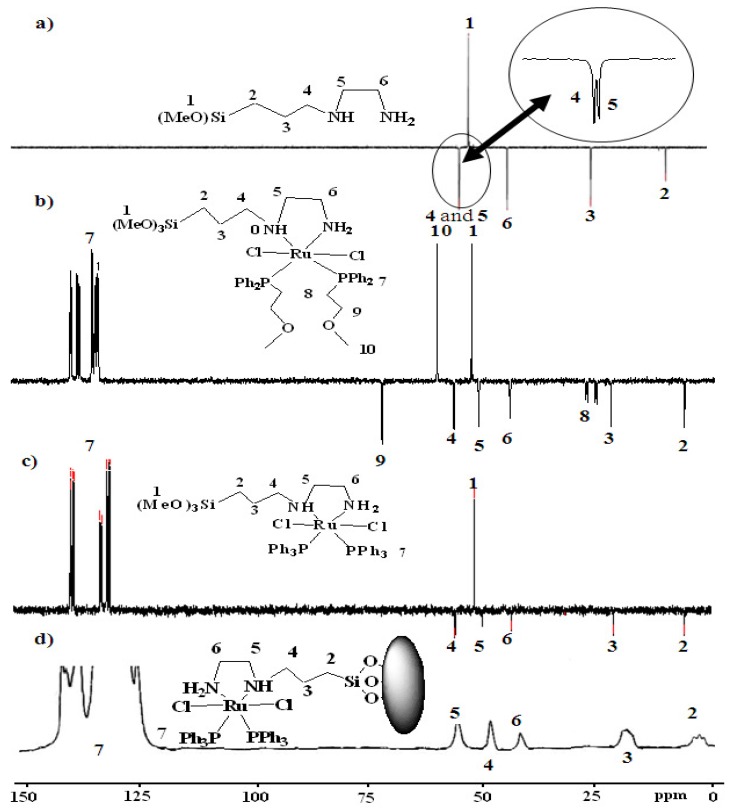
Dept 135 ^13^C-NMR of: a) free [3-(2-aminoethyl)aminopropyl]trimethoxysilane; b) complex **1** in CDCl_3_; c) complex **2** in CDCl_3_; compared by solid state ^13^C-CP-MAS-NMR; d)**X2** xerogel.

In the ^13^C-NMR spectra of complexes **1** and **2** and xerogels **X1** and **X2** characteristic sets of signals were observed, which are attributed to the PPh_3_ and P~O phosphine ligands as well as the [3-(2-amino-ethyl)aminopropyl]trimethoxysilane diamine co-ligand. Their assignment was supported by free ligand ^13^C-NMR studies. Several sets of aliphatic and aromatic carbons related to the phosphine and diamine were assigned with the help of 135 DEPT ^13^C-NMR to differentiate between the odd and even C-types, CH, CH_3_ up axis singlet, CH_2_ down axis singlet, and C no singlet. As a typical example, the 135 DEPT ^13^C-NMR spectra of the free [3-(2-aminoethyl)-aminopropyl]trimethoxysilane, complex **1**, complex **2**, and solid state ^13^C-CP-MAS-NMR of xerogels **X2** are shown in Figure 5.

Examination of the ^13^C-CP-MAS-NMR spectrum of the modified solids along with the solution phase spectrum of the corresponding molecular precursor led to the conclusion that the organic fragments in complex **2 **and xerogel **X2 ** remained intact during the grafting and subsequent workup without measurable decomposition (Figure 5d). The absence of the CH_3_O peak at δ_C_ = 49.92 belonging to (CH_3_O)_3_Si in the [3-(2-aminoethyl)aminopropyl]trimethoxysilane co-ligand after the sol-gel process of complex **2** to furnish xerogel **X2**, were the major differences noted between spectra, which supported the immobilization of the desired hybrid Ru(II) complexes. The total disappearance of groups in **X2** (Figure 5b), compared by complex **2** (Figure 5c and 5d), provides good confirmation of a sol-gel process gone to full completion [9,10,17].

**Figure 6:**
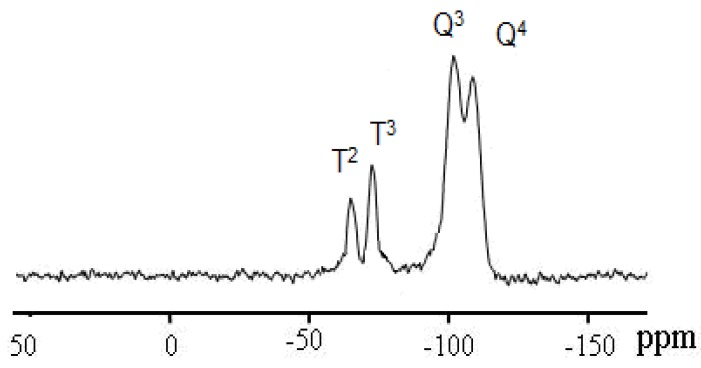
^29^Si CP/MAS NMR spectrum of **X2**, prepared by condensate of complex **2** with 10 equivalent of Si(OEt)_4_ cross-linkers.

Solid-state ^29^Si-NMR provided further information about the silicon environment and the degree of functionalization [9,10,17,27,28,29,30,31,32,33]. In all cases, the organometallic/organic fragment of the precursor molecule was covalently grafted onto the solid, and the precursors were, in general, attached to the surface of the polysiloxane by multiple siloxane bridges. The presence of T^m^ sites in case of xerogel **X1 ** and **X2 **in the spectral region of T^2^ at δ_Si_ = -56.8 ppm and T^3^ at δ_Si_ = - 69.1 ppm as expected, Q silicon sites due to Si(OEt)_4_ condensation agent were also recorded to Q^3^ at δ_Si_ = -101.2 and Q^4^ at δ_Si_ = -109.5 ppm silicon sites of the silica framework, as seen in Figure 6.

### 2.6. FAB mass spectra of the complex **1** and **2**

**Figure 7:**
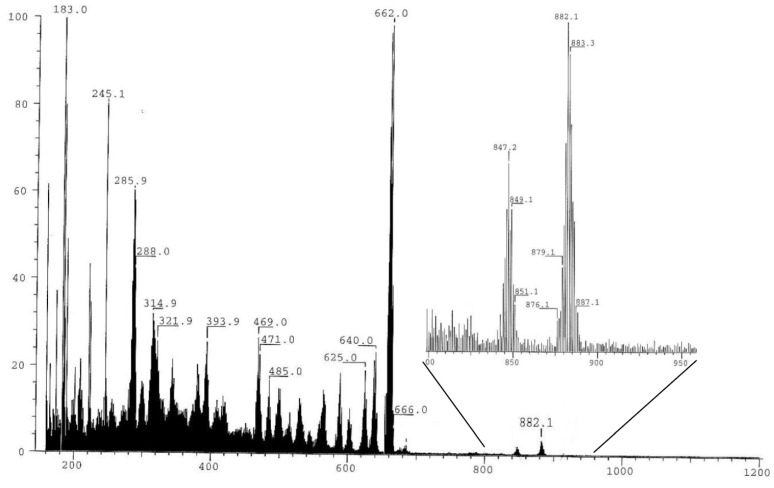
FAB-Mass spectrum of complex **1**.

Complexes **1** and **2** were subjected to FAB-MS, which showed molecular ion peaks M^+^ [Cl_2_Ru(PP)NN]^+^ at m/z 972.2 and 882.1 respectively, which revealed their exact calculated mass. For comparison the FAB-MS spectrum of complex **1** is presented in Figure 7. 

The first three fragments are the most important and were assigned as M^+^ = [Cl_2_Ru(P~O)2NN]^+^ at m/z 882.1, the second molecular ion was formed by the loss of HCl to give a fragment ion peak at m/z 847.2 belonging to M^+^-HCl =[RuCl_2_(P~O)2NN-HCl]^+^, the third fragment at m/z 662.0, which was the most stable one, was formed by loss of diamine ligand from the structure of complex **1** to give the starting material M^+^ -NN = [Cl_2_Ru(P^⏜^O)_2_]^+^, as seen in Figure 7.

### 2.7. IR investigations of ruthenium complexes **1** and **2**

**Figure 8:**
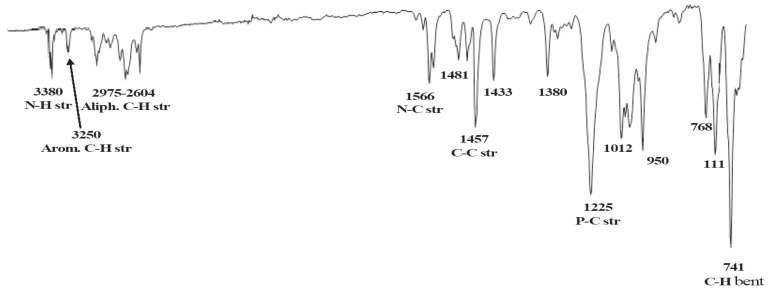
Infra-red spectrum cm^-1^ per well-known function group of complex **1**.

The IR spectra of the desired complexes in particular show several peaks which attributed to stretching vibrations of the main function group, in the ranges 3,490–3,300 cm^-1^ (*v*_NH_), 3,280–3,010 cm^‑1^ (*v*_PhH_) and 3,090–2,740 cm^-1 ^(*v*_CH_). All other characteristic bands due to the other function groups are also present in the expected regions, as seen in Figure 8. The IR spectrum which contained the chemical shifts of the main fragments represented the well-known function groups of complex **1** as an example was illustrated in Figure 8.

### 2.8. EXAFS measurement of xerogel X2

**Figure 9:**
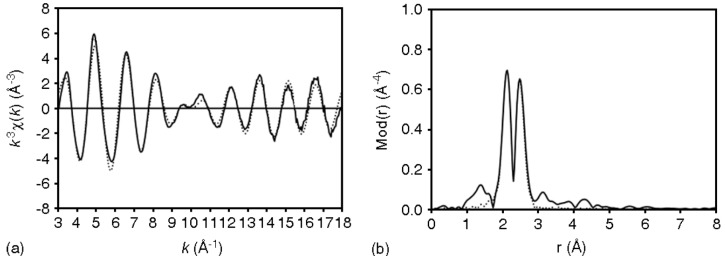
Experimental (solid line) and calculated (dotted line) EXAFS functions (a) and their Fourier transforms (b) for xerogel **X2** measured at Ru K-edge.

The xerogel **X2** was chosen as an example to determine the bond lengths between the metal center and the coordinating atoms of the ligand. The k^3^ weighted EXAFS function of xerogel **X2** can be described best by six different atom shells. The first intensive peak in the corresponding Fourier transform (Figure 9a) is mainly due to the nitrogen atoms. Chlorine and phosphorus atoms were found in the case of the most intense peak. For the most intense peak of the Fourier Transform, two equivalent phosphorus, two nitrogen atoms and two chlorine atoms with Ru-P, Ru-N and Ru-Cl bond distances of 2.27, 2.17 and 2.42 Å, respectively, were found (Figure 9b and Table 1). These results reveal a good agreement between the experimental and the calculated functions. 

**Table 1:** EXAFS determined structural parameters of xerogel **X2**.

A-Bs^a^	N^b^	r^c^ [Å]	σ^d^ [Å]	ΔE_0_^e^ [eV]	*k*-range [Å-^1^]	Fit-Index
Ru – N	2	2.17 ± 0.02	0.050 ± 0.005	22.26		22.06
Ru – P	2	2.27 ± 0.02	0.067 ± 0.007		3.0–18.0	
Ru – Cl	2	2.42 ± 0.03	0.054 ± 0.006			

^a ^absorber (A) – backscatterers (Bs), ^b^coordination number N, ^c^interatomic distance r, ^d^Debye-Waller factor σ with its calculated deviation and ^e^ shift of the threshold energy ΔE_0__._

### 2.9. Catalytic activity of complexes **1**, **2**, **X1** and **X2** in the hydrogenation of trans-4-phenyl-3-butene-2-al

**Scheme 2:**
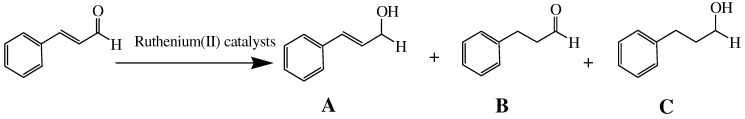
Different hydrogenation possibilities of *trans*-4-phenyl-3-butene-2-al:Selective carbonyl function group hydrogenation to produce **A**, selective C=C function group hydrogenation to produce **B**, full hydrogenation path with no selectivity to produce **C**.

To study the catalytic activity of the ruthenium(II) complexes, *trans*-4-phenyl-3-propene-2-al was selected, because three different rego-selective hydrogenation are expected (Scheme 2). The selective hydrogenation of the carbonyl group affords the corresponding unsaturated alcohol A. Unwanted and hence of minor interest both the hydrogenation of the C=C double bond, leading to the saturated aldehyde B and the full hydrogenation of C=O and C=C bonds resulting the formation of saturated alcohol C. The hydrogenation reactions using complexes **1** and **2**, and xerogel **X1** and **X2** as catalysts were carried out under identical conditions: 35 °C with a molar substrate:catalyst (TON, S/C) ratio of 1,000:1, under 2 bar of hydrogen pressure, in 50 mL of 2-propanol [Ru: Co-catalysts (KOH, tBuOK and K_2_CO_3_): *trans*-4-phenyl-3-butene-2-al] [1:10:1,000], the results are listed in Table 2.

**Table 2:** Hydrogenation of *trans*-4-phenyl-3-butene-2-al by Ru(II) complexes.

Run	Catalyst	Co-catalyst	Conversion (%)^a^	Selectivity (%) ^a^	TOF ^b^
1	**1**	*t*BuOK	>99 ^c^	>99 **A**	1,160
2	**2**	*t*BuOK	>99^ c^	>99 **A**	1,050
3	**1**	KOH	>99^ c^	>99 **A**	1,210
4	**2**	KOH	>99^ c^	>99 **A**	1,070
5	**1 **or** 2**	K_2_CO_3_	0^ d^	-	-
6	**X1**	*t*BuOK	95^ d^	93 **A**	80
7	**X2**	*t*BuOK	90^ d^	90 **A**	75
8	**X1**	KOH	94^ d^	92 **A**	78
9	**X2**	KOH	92^ d^	91 **A**	76

^a ^Yield and selectivity were determined by GC. ^b ^Turnover frequency: mole of product per mole of catalyst per hour, h^-1^. ^c^ The reaction was conducted to the hydrogenation for one hour. ^d^ The reaction was conducted to the hydrogenation for 12 hours

These catalysts were only effective in the presence of excess hydrogen in 2-propanol and a strong basic co-catalyst like KOH and*t*BuOK, since when weakly basic K_2_CO_3_ was used as co-catalyst, no hydrogenation reaction was observed, even after longer reaction times. Complexes **1** and **2** are highly active under these mild conditions and gave rise to 99% conversion and selective hydrogenation of the C=O group in the presence of a C=C function. Complex **2** was slightly less active under identical conditions compared to complex **1**, which can be attributed to the hemilability of the ether-phosphine ligand in the diamine/ruthenium(II) system.

**Figure 10:**
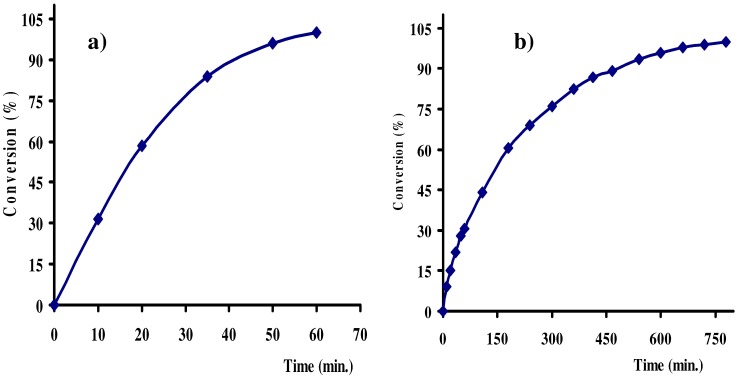
Hydrogenation reaction of *trans*-4-phenyl-3-butene-2-al using complex **1** and xerogel **X1** catalysts under the above mild conditions.

The other ruthenium(II) precursors, xerogels **X1** and **X2**, displayed high conversion ratios and selectivity (~ 90%) in the C=O selective hydrogenation of the *trans*-4-phenyl-3-butene-2-al using strong basic conditions. Expected constant decrease in the activity and the selectivity were observed by comparing the homogenous **1** and **2** with the heterogeneous **X1** and **X2** precursors under identical conditions. The hydrogenation reaction under the above conditions using complex **1** as catalyst was finished within one hour, as seen in Figure 10a, while xerogel **X1** under the same condition takes ~ 12 hours to react to 95% conversion, as evident in Figure 10b. The GC-conversion of the hydrogenation process was plotted *vs.* reaction time in minutes as illustrated in Figure 10. 

## 3. Experimental

### 3.1. General

All reactions were carried out in an inert atmosphere (argon) by using standard high vacuum and Schlenk-line techniques, unless otherwise noted. Prior to use CH_2_Cl_2_, *n*-hexane, and Et_2_O were distilled from CaH_2_, LiAlH_4_, and from sodium/benzophenone, respectively, ether-phosphine ligand, Cl_2_Ru(P^⏜^O)_2 _and Cl_2_Ru(PPh_3_)_2_ were prepared according to literature methods [9,10,11,12,13]. [3-(2-aminoethyl)aminopropyl]trimethoxysilane and tetramethoxysilyl were purchased from Acros. Elemental analyses were carried out on an Elementar Vario EL analyzer. High-resolution liquid ^1^H-, ^13^C{^1^H}-, DEPT 135, and ^31^P{^1^H}-NMR spectra were recorded on a Bruker DRX 250 spectrometer at 298 K. Frequencies are as follows: ^1^H-NMR: 250.12 MHz, ^13^C{^1^H}-NMR: 62.9 MHz, and ^31^P{^1^H}-NMR 101.25 MHz. Chemical shifts in the ^1^H- and ^13^C{^1^H- NMR spectra were measured relative to partially deuterated solvent peaks which are reported relative to TMS. ^31^P-NMR chemical shifts were measured relative to 85% H_3_PO_4_. CP/MAS solid-state NMR spectra were recorded on Bruker DSX 200 (4.7 T) and Bruker ASX 300 (7.05 T) multinuclear spectrometers equipped with wide-bore magnets. Magic angel spinning was applied at 4 kHz (^29^Si) and 10 kHz (^13^C, ^31^P) using (4 mm ZrO_2_ rotors). Frequencies and standards: ^31^P, 81.961 MHz (4.7 T), 121.442 MHz (7.05 T) [85% H_3_PO_4_, NH_4_H_2_PO_4_ (δ = 0.8) as second standard]; ^13^C, 50.228 MHz (4.7 T), 75.432 MHz (7.05 T) [TMS, carbonyl resonance of glycine (δ = 176.05) as second standard]; ^29^Si, 39.73 MHz (4.7 T), 59.595 MHz (7.05 T, (Q8M8 as second standard). All samples were prepared with exclusion of molecular oxygen. . IR data were obtained on a Bruker IFS 48 FT-IR spectrometer. Mass spectra: EI-MS, Finnigan TSQ70 (200 °C) and FAB-MS, Finnigan 711A (8 kV), modified by AMD and reported as mass/charge *(m/z). *The analyses of the hydrogenation experiments were performed on a GC 6000 Vega Gas 2 (Carlo Erba Instrument) with a FID and capillary column PS 255 [10 m, carrier gas, He (40 kPa), integrator 3390 A (Hewlett Packard)]. The EXAFS measurements were performed at the ruthenium K–edge (22118 eV) at the beam line X1.1 of the Hamburger Synchrotronstrahlungslabor (HASYLAB) at DESY Hamburg, under ambient conditions, energy 4.5 GeV, and initial beam current 120 mA. For harmonic rejection, the second crystal of the Si(311) double crystal monochromator was tilted to 30%. Data were collected in transmission mode with the ion chambers flushed with argon. The energy was calibrated with a ruthenium metal foil of 20 μm thickness. The samples were prepared of a mixture of the samples and polyethylene.

### 3.2. General procedure for the preparation of the complex **1** and **2**

Diamine (0.23 mmol, 5% excess) was dissolved in dichloromethane (5 mL), the solution was added dropwise to a stirred solution of Cl_2_Ru(P^⏜^O)_2_ and Cl_2_Ru(PPh_3_)_3_(0.22 mmol) in dichloromethane (10 mL) within 2 min. The mixture was stirred for ca. 2 h at room temperature while the color changed from brown to yellow, then the volume of the solution was concentrated to about 2 mL under reduced pressure. Addition of diethyl ether (40 mL) caused the precipitation of a solid which was filtered (P4), then dissolved again in dichloromethane (40 mL) and concentrated again under vacuum to a volume of 5 mL. Addition of n-hexane (80 mL) caused the precipitation of a solid which was filtered (P4), well washed with *n*-hexane each and dried under vacuum. Complex **1 ** and **2** were obtained in analytically pure form in very good yields. m.p. > 340 °C (dec.). *Complex* **1**: ^1^H-NMR (CDCl_3_): δ (ppm) 0.11 (m, 2H, CH_2_Si), 0.57 (m, 2H, SiCH_2_C*H_2_*), 1.45 (br, 2H, SiCH_2_CH_2_C*H_2_*N), 2.02 (m, 2H, CH_2_N*CH*_2_CH_2_N), 2.34 (m, 2H, CH_2_N*C*H_2_C*H_2_*N), 2.44 (br, 4H, PC*H_2_*), 2.88, 2.92 (2s, 6H, C*H_3_*OCH_2_), 3.08 (m, 4H, CH_2_O), 3.48 (br, 9H, CH_3_OSi), 3.55 (s, 3H, NH_2_), 6.90–7.90 (m, 20H, C_6_H_5_); ^31^P{^1^H}-NMR (CDCl_3_): δ (ppm) 35.64, 38.87, dd, AB pattern with *J*pp = 35.6 Hz, ^13^C{^1^H}-NMR (CDCl_3_): δ (ppm) 6.67 (s, C, CH_2_Si), 21.11 (s, SiCH_2_*C*H_2 _), 26.12, 27.03 (2m, 2C, PCH_2_), 43.82 (s, C, HNCH_2_*C*H_2_NH_2_), 48.43 (s, C, HN*C*H_2_CH_2_NH_2_), 50.32 (s, 3C, SiOCH_3_), 55.45 (s, C, SiCH_2_CH_2_*C*H_2_NH), 57.93, 58.01 (2s, 2C, OCH_3_), 69.31, 69.40 (2s, 2C, OCH_2_), 127.20-134.0 (m, 24C, C_6_H_5_); FAB–MS; *(m/z)*: 882.2 (M^+^); Yield 92% related to Ru(II); Anal. Calc. C, 51.70; H, 6.39; Cl, 8.03; N, 3.17; for C_38_H_56_Cl_2_N_2_O_5_P_2_RuSi: Found C, 51.55; H, 6.24; Cl, 8.23; N, 3.54 %. *Complex ***2: **^1^H-NMR (CDCl_3_): δ (ppm) 0.12 (m, 2H, CH_2_Si), 0.82 (m, 2H, SiCH_2_C*H_2_*), 1.32 (br, 2H, SiCH_2_CH_2_C*H_2_*N), 2.41 (m, 2H, CH_2_N*CH*_2_CH_2_N), 2.81 (m, 2H, CH_2_N*C*H_2_C*H_2_*N), 3.29 (s, 3H, NH_2_), 3.46 (br, 9H, CH_3_OSi), 6.90-7.60 (m, 30H, C_6_H_5_); ^31^P{^1^H}-NMR (CDCl_3_): δ (ppm) 39.9 , 44.1 dd, AB pattern with *J*pp = 31.6 Hz, ^13^C{^1^H}-NMR (CDCl_3_): δ (ppm) 6.69 (s, C, CH_2_Si), 21.82 (s, SiCH_2_*C*H_2 _), 43.01 (s, C, HNCH_2_*C*H_2_NH_2_), 49.17 (s, C, HN*C*H_2_CH_2_NH_2_), 50.86 (s, 3C, SiOCH_3_), 54.92 (s, C, SiCH_2_CH_2_*C*H_2_NH), 127.20-135.60 (3m, 36C, C_6_H_5_); FAB–MS; *(m/z)*: 918.1 (M^+^); Yield 83% related to Ru(II); Anal. Calc. C, 57.25; H, 7.01; Cl, 7.59; N, 3.08; for C_48_H_58_Cl_2_N_2_O_3_P_2_RuSi: Found C, 57.51; H, 5.70; Cl, 7.72; N, 3.05%.

### 3.3. General procedure for sol–gel processing of xerogels **X1** and **X2**

Complexes**1 **and **2** (0.100 mmol) and Si(OEt)_4_ (1 mmol,10 equivalents) in THF (5 mL) were mixed together. The sol–gel took place when a methanol/water mixture (2 mL, 1:1 v/v) was added to the solution. After 24 h stirring at room temperature, the precipitated gel was washed with toluene and diethyl ether (30 mL of each), and petroleum ether (20 mL). Finally the xerogel was ground and dried under vacuum for 24 h to afford after workup ~ 300 mg [yield ~45% based on Ru(II)] of a pale yellow powder were collected.

*Xerogel* **X1**: ^31^P-CP/MAS-NMR: δ = 35.64, 38.87, dd, AB pattern with *J*_pp_ = 35.6 Hz; ^13^C-CP/MAS NMR: δ (ppm) 5.44 (br, 1C, CH_2_Si), 20.65 (m, 1C, *C*H_2_CH_2_Si), 27.25 (m, 2C, PCH_2_), 43,87 (br, 1C, NH_2_*C*H_2_CH_2_NH), 48.32 (br, 1C, NH_2_CH_2_), 55.72 (s, 1C, NH*C*H_2_CH_2_CH_2_), 57.21 (br, 2C, OCH_3_), 69.77 (br, 2C, OCH_2_), 120.00-140.00 (m, 24C, C_6_H_5_); ^29^Si CP/MAS NMR: δ = –67.2 ppm (T^3^), –57.4 ppm (T^2^), -101.8 ppm (Q^3^), -109.3 ppm (Q^4^).

*Xerogel* **X2**: ^31^P-CP/MAS-NMR: δ = 39.9 , 44.1 ppm. dd, *J*_pp_ =31.6 Hz; ^13^C-CP/MAS NMR: δ (ppm) 6.68 (br, 1C, CH_2_Si), 22.12 (m, 1C, *C*H_2_CH_2_Si), 42.31 (s, 1C, NH_2_*C*H_2_CH_2_NH), 48.44 (br, 1C, NH_2_CH_2_), 54.82 (s, 1C, NH*C*H_2_CH_2_CH_2_), 120.00-140.00 (m, 36C, C_6_H_5_); ^29^Si CP/MAS NMR: δ = –69.1 ppm (T^3^), –56.8 ppm (T^2^), -101.2 ppm (Q^3^), -109.5 ppm (Q^4^).

## 4. Conclusions

Four new diamine/phosphine/ruthenium(II) complexes were prepared. Complexes **1** and **2** were prepared by ligand exchange and hemilable cleavage methods, respectively. The presence of T-silyl functions on the diamine co-ligand backbone enabled the hybridization of these complexes in order to support them on a polysiloxane matrix through the sol-gel technique in order to produce xerogels **X1** and **X2**. The structural behaviors of the phosphine ligands in the desired complexes during synthesis were monitored by ^31^P{^1^H}-NMR. The structure of complexes **1** and **2** described herein have been deduced from elemental analyses, infrared, FAB-MS and ^1^H-, ^13^C-, H, and ^31^P-NMR spectroscopy data. The xerogel structuresof **X1** and **X2** were determined by solid state ^13^C-, ^29^Si- and ^31^P-NMR spectroscopy, infrared spectroscopy and EXAFS. When these complexes were tested as catalysts for the hydrogenation of *trans*-4-phenyl-3-butene-2-al in both homogenous and heterogeneous phases, they showed a high degree of stability and activity as well as an excellent degree of carbonyl hydrogenation selectivity under mild conditions.
